# Protein Arginine Methyltransferases in Neuromuscular Function and Diseases

**DOI:** 10.3390/cells11030364

**Published:** 2022-01-21

**Authors:** Jinwoo Lee, Subin An, Sang-Jin Lee, Jong-Sun Kang

**Affiliations:** 1 Research Institute for Aging-Related Diseases, AniMusCure Inc., Suwon 16419, Korea; jwlee@animuscure.com; 2 Department of Molecular Cell Biology, School of Medicine, Sungkyunkwan University, Suwon 16419, Korea; thdrlwk02@hanmail.net; 3 Single Cell Network Research Center, Sungkyunkwan University, Suwon 16419, Korea

**Keywords:** PRMT, neuromuscular diseases, ALS, muscle atrophy

## Abstract

Neuromuscular diseases (NMDs) are characterized by progressive loss of muscle mass and strength that leads to impaired body movement. It not only severely diminishes the quality of life of the patients, but also subjects them to increased risk of secondary medical conditions such as fall-induced injuries and various chronic diseases. However, no effective treatment is currently available to prevent or reverse the disease progression. Protein arginine methyltransferases (PRMTs) are emerging as a potential therapeutic target for diverse diseases, such as cancer and cardiovascular diseases. Their expression levels are altered in the patients and molecular mechanisms underlying the association between PRMTs and the diseases are being investigated. PRMTs have been shown to regulate development, homeostasis, and regeneration of both muscle and neurons, and their association to NMDs are emerging as well. Through inhibition of PRMT activities, a few studies have reported suppression of cytotoxic phenotypes observed in NMDs. Here, we review our current understanding of PRMTs’ involvement in the pathophysiology of NMDs and potential therapeutic strategies targeting PRMTs to address the unmet medical need.

## 1. Introduction

Loss of muscle mass and strength leads to declined functional capacity and greater risk of developing chronic diseases that include neuromuscular diseases, cancer, inflammatory diseases, and diabetes [1,2,3,4]. In turn, many chronic diseases often result in muscle weakness and atrophy [4]. Accumulating evidence suggests that the maintenance of muscle health is critical to reduce the prevalence of chronic diseases, thereby increasing healthy life span [4]. Thus, many efforts have been made to understand the underlying molecular mechanisms of muscle metabolism and function to develop strategies to enhance muscle health [5]. Perturbations in muscle regenerative capacity, muscle mitochondrial function, balance in the protein synthesis/degradation pathway and neuromuscular activity have been associated with muscle atrophy and weakness [6]. In this review, we will focus on the recent advances in understanding the functional significance of protein arginine methyltransferases (PRMTs) in muscle atrophy and neuromuscular function with a specific emphasis on the potential role of arginine methylation in neuromuscular diseases (NMDs).

## 2. Protein Arginine Methyltransferases (PRMTs)

Post-translational modifications such as phosphorylation, ubiquitination, and methylation have been shown to play a critical role in diverse biological processes such as gene transcription, mRNA splicing, DNA repair, signaling transduction, protein subcellular localization, and cell cycle progression. PRMTs are a family of enzymes that catalyze the transfer of a methyl group from S-adenosyl-L-methionine (AdoMet) to L-arginine on target proteins, thereby altering the stability, localization, and/or activity of the marked molecules [7]. PRMTs consist of nine members and are classified into three categories according to their catalytic activities: type I (PRMT1, PRMT2, PRMT3, PRMT4, PRMT6, and PRMT8), type II (PRMT5 and PRMT9), and type III (PRMT7). Type I and Type II PRMTs perform the formation of mono-methylarginine (MMA) as an intermediate step before the establishment of asymmetric dimethylarginine (ADMA) or symmetric dimethylarginine (SDMA), respectively [8,9,10]. Type III PRMT only carries out the formation of MMA [11]. PRMT1 is the predominant PRMT in mammalian cells and performs at least 85% of all arginine methylation activity in human cells [12]. The most common sites that are targeted by PRMTs are arginine and the glycine-rich motif termed the RGG/RG motif [13]. While the presence of glycine next to arginine facilitates the activity of PRMTs, it is not a requirement, as there are many exceptions. The existence of enzymes capable of demethylating methylarginines is central to the concept that arginine methylation is a dynamic modification [14]. Two types of enzymes, peptidylarginine deiminase type IV (PAD4) and Jumonji domain-containing (JMJDs), have been reported to remove methyl groups from arginine residues in proteins [15,16]. However, the biological significance of both enzymes in regulating protein arginine methylation in vivo is yet to be confirmed.

**Figure 1:**
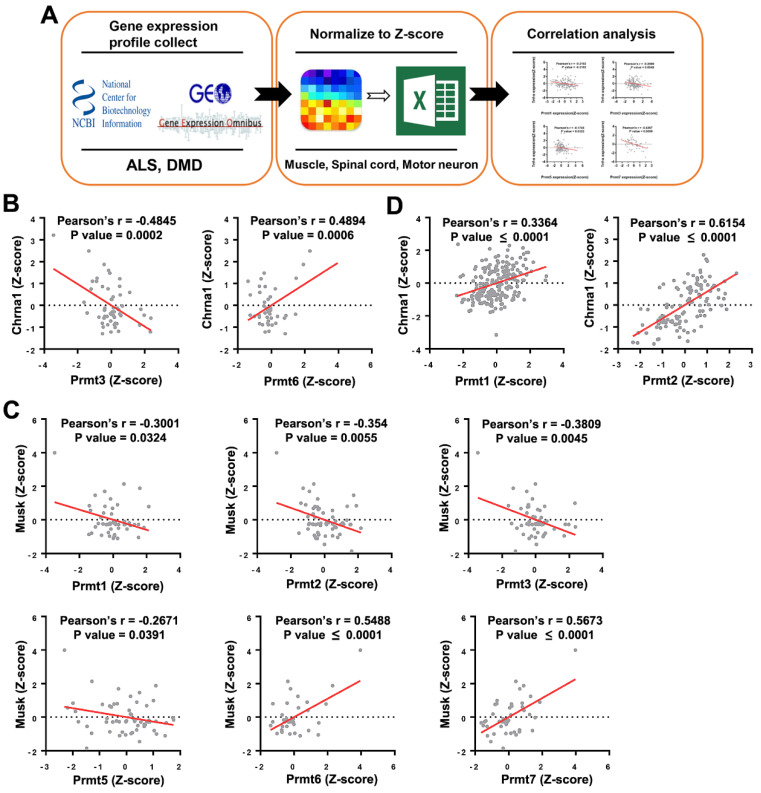
Correlation between expression of Prmts and NMJ markers in datasets of amyotrophic lateral sclerosis (ALS) and Duchenne muscular dystrophy (DMD) disease models obtained from an open database. (**A**) A schematic illustration demonstrating the stepwise workflow of the transcriptome analysis. Gene expression profile of muscle, spinal cord, and motor neuron from various species was collected from the Gene Expression Omnibus of the National Center for Biotechnology Information; the value of gene expression was normalized to Z-score, and correlation between Prmts and NMJ markers was analyzed. (**B**,**C**) Scatter plots presenting the correlated expression patterns between Prmts (X-axis) and NMJ markers (Y-axis) in ALS model. Cholinergic Receptor Nicotinic Alpha. (**D**) Correlated expression patterns between PRMTs (X-axis) and Chrna1 (Y-axis) in DMD. (ALS: n = 73, DMD: n = 255).

**Figure 2:**
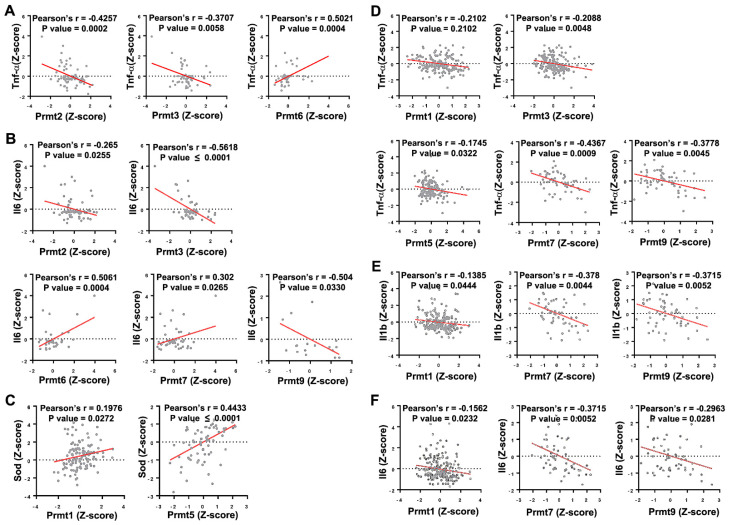
Correlation between Prmts and oxidative stress markers in ALS and DMD models. (**A**,**B**) Scatter plots presenting the correlated expression patterns between Prmts (X-axis) and oxidative stress markers (Y-axis) in ALS model. Tumor Necrosis Factor-Alpha (Tnf-α) (**A**), Interleukin 6 (Il6) (**B**). (**C**–**F**) Correlated expression patterns between Prmts (X-axis) and oxidative stress markers (Y-axis) in DMD model. Superoxide Dismutase (Sod) (**C**), Tnf-α (**D**), Interleukin 1 Beta (Il1b) (**E**), Il6 (**F**). (ALS: n = 71, DMD: n = 255).

PRMTs are generally ubiquitously expressed and dysregulated expression or activity has been implicated in the progression of several prevalent health conditions, such as cancer and cardiovascular disease [17]. The association between PRMTs and NMDs is also emerging (Figure 1 and Figure 2). In mice, knockout (KO) of PRMT1 or PRMT5 has shown to be embryonic lethal [18,19], while KO of other PRMT isoforms has shown varying phenotypes from neonatal death to a developmental delay without further abnormalities [20,21,22]. The severe phenotype following whole body KO of PRMTs led to studies utilizing tissue-specific PRMT null animals to investigate the role of each PRMT isoform. Due to there being more than thousands of substrates with both functional redundancy and substrate specificity, the delineation of a physiological role of each PRMT isoform has been quite a challenge [23]. Regardless, the in vivo studies continue to support the clinical relevance of PRMTs [14,24,25]. A more detailed description of PRMTs’ structure and function can be found in the following reviews [8,14,24,26,27].

## 3. PRMTs in Muscle Homeostasis and Remodeling

PRMTs are expressed in both skeletal muscle and muscle satellite cells (MSCs) [28,29], and they have been shown to regulate skeletal muscle plasticity, specifically myogenesis. MSC-specific PRMT1 deficient mice show compromised muscle repair following cardiotoxin (CTX)-induced injury [30]. The authors further identified Eya1 and Six1, which are coactivators of MyoD, as the PRMT1 substrates, suggesting that PRMT1 regulate muscle stem cell fate via Eya1/Six1/MyoD axis [30]. Arginine methylation of Pax7 by PRMT4 functions as a molecular switch controlling the induction of Myf5 during satellite cell asymmetric division and entry into the myogenic program [31,32]. Work investigating the role of PRMT5 in MSCs found that PRMT5 generates a ready state that keeps MSCs in standby, allowing rapid amplification when needed [33,34]. PRMT7 has been shown to be a regulator of the DNMT3b/p21 axis, which is required to maintain MSC regenerative capacity [35]. Also, PRMT7 promotes MyoD-mediated myogenic differentiation through methylation of arginine residue 70 of p38MAPK [36]. As a result, conditional KO of PRMT4, PRMT5, or PRMT7 in MSCs all led to compromised muscle regeneration following CTX-induced injury [31,33,35].

In skeletal muscle cells, PRMTs are implicated in muscle remodeling by controlling autophagy and muscle metabolism. Muscle-specific KO of PRMT1 causes muscle atrophy through upregulation of the PRMT6/FOXO3 axis that enhances autophagy [37]. PRMT4 is upregulated following nutrient starvation and promotes autophagy by methylating FOXO3 and acting as a co-activator of transcription factor EB to enrich autophagy-related and lysosomal genes [38,39]. Several in vitro studies on skeletal muscle cells have proposed a potential role of PRMT1 and PRMT4 in muscle metabolism as well. PRMT1 regulates the insulin receptor/insulin receptor substrate-1/phosphatidylinositol 3-kinase pathway, which is involved in glucose transport [28]. PRMT4 is necessary for the expression of genes involved in glycogen metabolism in skeletal muscle cells [28,40]. PRMT7 is a key regulator of slow, oxidative myogenic program by enhancing peroxisome proliferator activated receptor-γ coactivator-1α (PGC1α) expression through the p38/ATF2/PGC1α pathway [41]. Whole body PRMT7 KO animals exhibit decreased oxidative metabolism and attenuated endurance exercise capacity compared to their wild type littermates. While further studies will be necessary to define the role of other members of the PRMT family in muscle homeostasis and remodeling, PRMTs have emerged as the critical regulator of the process and an intriguing target for treating NMDs.

## 4. PRMTs in Neuromuscular Function

The nervous system and muscular system are tightly connected through a specialized junction called the neuromuscular junction (NMJ). NMJ is a synapse that forms between motor neurons and skeletal muscle and is covered by terminal Schwann cells. During development, the nerve terminal of motor neurons releases agrin, which binds to the agrin receptor Lrp4 of the myotube. This activates transcription of a set of genes necessary for acetylcholine receptor (AChR) cluster formation at the post-synaptic part of the myotube [42]. In turn, muscle cells release signaling proteins such as beta-catenin that are required for the pre-synaptic differentiation of the motor neurons [43]. When a body movement is triggered, lower motor neurons release acetylcholine (Ach) into the synaptic cleft, and the muscle membrane detects this chemical signal via AChRs and triggers muscle contraction by activating voltage-gated dihydropyridine receptors and ryanodine receptors in the sarcolemma [42]. As a result, the motor neurons, myotubes, and NMJs constitute an intricate functional unit for our body movement, and a problem that occurs in one part severely affects the rest of the unit.

PRMTs are expressed strongly in the entire nervous central nervous system (CNS) [44] and have shown to be critical for the maintenance of neuromuscular function. The conditional KO of PRMT1 in the neural stem cells leads to the neonatal death of mice, displaying a severe defect in the myelination of neurons [45]. The deletion of PRMT5 in neural stem cells also leads to neonatal death [46], and the enzyme is suggested to be involved in maintaining a proliferative state of neural stem cells via symmetric dimethylation of histone H4 arginine 3 [47]. Both PRMT1 and PRMT5 were shown to be indispensable for oligodendrocyte differentiation and myelination [45,48]. PRMT4 is also implied in the maintenance of a proliferative state of neural progenitor cells through methylation of HuD, an RNA binding protein (RBP), that disrupts its binding to p21cip1/waf1 [49]. Also, PRMT4 regulates astroglial cell fate via histone H3 arginine 17 methylation [50]. PRMT8 in the brain regulates synapse maturation, and the brain-specific loss of PRMT8 leads to compromised hippocampus-dependent memory [51,52]. Studies have also revealed that PRMT1 and PRMT7 regulate ion channel activity and the excitability of neurons [53,54]. Since PRMTs are involved in differentiation, myelination, synapse formation, and the activity of neurons, it is unsurprising that alteration in PRMT activities is associated with neurodegeneration and NMDs [14]. 

## 5. PRMTs in Neuromuscular Diseases (NMDs)

NMDs are characterized by impaired skeletal muscle due to degeneration of motor neurons, NMJs, or muscle cells. NMDs are categorized based on the site of origin and whether the cause is inherited or sporadic [55]. Some of the common NMDs include amyotrophic lateral sclerosis (ALS), multiple sclerosis (MS), myasthenia gravis (MG), and Duchenne muscular dystrophy (DMD). Mounting evidence suggests that PRMT is a major player in the etiology of NMDs. PRMTs have been shown to interact with many proteins associated with NMDs and modulation of PRMT activities has shown to diminish pathological features in cells. Post-mortem spinal cords of ALS patients show higher immunoreactivity against PRMT1 as well as higher concentrations of ADMA in the cerebrospinal fluid compared to those of the control group [56], further supporting the relevance of PRMTs in disease progression. However, how PRMTs are involved in the etiology of NMDs remains unresolved, and mechanistic studies are actively ongoing. This section will explore three mechanisms of PRMTs involvement in the progression of NMDs.

### 5.1. Regulation of Cytoplasmic Ribonucleoprotein (RNP) Granules

**Figure 3:**
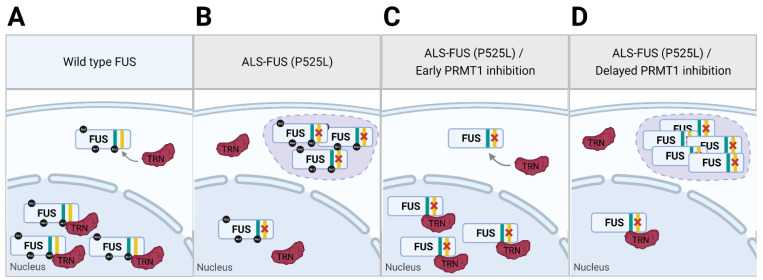
Dual function model of PRMT1 in cells expressing ALS-associated FUS mutant. (**A**) Wild type FUS is transported to the nucleus by transportin (TRN) that binds to NLS (yellow) of FUS. (**B**) Mutation (P525L) in ALS-FUS disrupts the interaction between NLS and TRN, leading to cytoplasmic accumulation of ALS-FUS and formation of RNP granules. (**C**) PRMT1 inhibition that precedes ALS-FUS expression reveals alternative binding site of FUS (cyan) to TRN, rescuing the nuclear transport of ALS-FUS. (**D**) Concurrent PRMT1 inhibition with ALS-FUS expression fails to rescue the nuclear transport of already mis-localized ALS-FUS. Reduced methylation of cytoplasmic FUS by PRMT1 inhibition strengthens π-cation interaction of ALS-FUS, exacerbating ALS phenotype.

Cytoplasmic accumulation of protein aggregates is a pathological hallmark of various neurodegenerative diseases such as ALS, frontotemporal dementia, Alzheimer’s diseases, and Huntington’s diseases. ALS is a severe neuromuscular disease characterized by the loss of both upper and lower neurons, leading to muscle atrophy, stiffening, and paralysis. It is one of the most common degenerative diseases of motor neurons with incidence rates of 0.6–3.8 per 100,000 individuals, and it usually causes death within three to five years after the beginning of symptoms in ALS patients, mostly due to respiratory failure [57,58,59]. However, the etiology of the disease is not well understood, and thus, no effective cure is currently available. Approximately 90% of cases are classified as sporadic ALS (sALS), while 8 to 10% are categorized as familial ALS (fALS) [58]. In post-mortem brain and spinal cord of both sALS and fALS patients, cytoplasmic fused in sarcoma (FUS)-positive or TAR DNA-binding-43KDa (TDP-43)-positive inclusions are found in over 90 percent of the cases [60]. FUS and TDP-43 are ubiquitously expressed RBPs involved in the processing and transporting of RNAs. In neurons and glial cells, FUS is predominantly localized in the nucleus and plays a role in dendritic spine formation, RNA transport, mRNA stability, and synaptic homeostasis [61]. It contains tyrosine-rich low-complexity domain (LCD) in the N-terminal domain, an RNA-recognition motif, multiple RGG domains, and a nuclear localization signal (NLS) in the C-terminal domain [62]. The NLS of FUS has shown to be required for the nuclear import of FUS, and it is dependent on the interaction with transportin (Figure 3A) [63,64]. ALS-associated mutations of FUS are mostly found within the NLS [65,66], impairing the nuclear import of FUS and causing cytoplasmic accumulation of the protein (Figure 3B). TDP-43 contains an NLS in the N-terminal domain, two RNA-recognition motifs, followed by a glycine-rich LCD [67]. Unlike FUS, over 30 ALS-associated mutations of TDP-43 do not affect the NLS [63]. Studies suggest that the mutations rather promote aggregation propensity of TDP-43 in the cytoplasm [68,69]. The cytoplasmic accumulation of the RBPs increases local protein concentration and triggers liquid-liquid phase separation (LLPS) of the RBPs to form a reversible membraneless organelle. In a non-pathological condition, these transient RNP granules are suggested to be beneficial through the silencing of RNA translation in response to stress or by transporting RNAs to cell extremities to perform localized translation [70]. However, persistent RNP granules become fibrotic and cytotoxic.

Studies suggest that PRMTs may be involved in both nuclear transport and LLPS of the RBPs. FUS contains over 20 dimethyl arginine residues, which are mainly found in the RGG3 domain nearby NLS [71], and the residues have been shown to be targeted by PRMT1 and PRMT8 [72,73]. Treating cells with the general methylation inhibitor adenosine-2,3-dialdehyde (AdOx), restores the nuclear import of ALS-associated FUS mutants (ALS-FUS) [64,72,73]. PRMT1 knockdown also diminishes the cytoplasmic accumulation of ALS-FUS [64,72,73]. Dormann et al. showed that this rescue of ALS-FUS phenotype by AdOx treatment requires the presence of arginines in the RGG3 domain of FUS [64]. The authors further demonstrated that the unmethylated RGG3 domain of ALS-FUS binds to transportin, while methylation of the RGG3 domain prevents this binding. Based on these data, Dormann et al. proposed a model describing an alternative binding site of FUS to transportin in the RGG3 domain, which is only unmasked when the methylation is removed (Figure 3C). This model elegantly explains how the inhibition of methyltransferase activity or PRMT1 knockdown can rescue the nuclear import of ALS-FUS in cells. The model is further supported by an independent study showing much higher affinity of unmethylated or monomethylated FUS to transportin than asymmetrically dimethylated FUS [74]. In the post-mortem tissues of ALS patients, unmethylated or monomethylated FUS are not found readily [74], which is in line with the proposed function of PRMT1 in the cytoplasmic accumulation of ALS-FUS.

What drives LLPS of proteins and RNAs in cells is still a topic under investigation. In the case of FUS, LLPS is suggested to be driven by the electrostatic force between the pi electron cloud of the aromatic rings in the tyrosine-rich LCD and cations of arginine and lysine residues. This electrostatic force is termed the π-cation interaction [75,76]. Therefore, the tyrosine-rich LCD and RGG domains of FUS have been found to be necessary for the LLPS. Of note, arginine residues outside of RGG domains can also participate in π-cation interactions, and the necessity of RGG domains is simply due to the arginine-rich nature of the domains. The methylation of arginine introduces bulkiness and hydrophobicity while maintaining the charge of the residue [77]. In the context of π-cation interactions, arginine methylation has been shown to weaken the interaction, and thus, suppress LLPS of FUS [78,79,80,81]. This complicates the delineation of PRMTs’ role in cytoplasmic RNP granule formation. While methylation of ALS-FUS prevents the RGG3 domain’s interaction with transportin, which leads to cytoplasmic accumulation of the protein, it can suppress LLPS by weakening π-cation interactions once the proteins are mislocalized to the cytoplasm.

This dual role of PRMT in cytoplasmic RNP granule formation means that the consequences of PRMT inhibition can vary based on the timing and context of the inhibition. Tradewell et al. showed that while AdOx treatment or PRMT1 knockdown at least 48 h prior to the transfection of ALS-FUS leads to diminished cytoplasmic ALS-FUS, concurrent PRMT1 knockdown alongside the transfection of ALS-FUS leads to an increase in cytoplasmic ALS-FUS [72]. This opposing effect of PRMT1 knockdown can be explained by the delayed knockdown of PRMT1 not having enough time to rescue the nuclear import of ALS-FUS, but in time to promote the LLPS of ALS-FUS and exacerbate the mutant phenotype (Figure 3D). A study by another group also found increased cytoplasmic ALS-FUS by PRMT1 knockdown in cortical neurons, and the knockdown was performed by concurrent transfection of PRMT1 shRNA and ALS-FUS plasmids [82]. In vivo studies in flies by two independent groups have demonstrated that knockdown of the PRMT1 ortholog, Dart1, exacerbates the neurodegeneration phenotype induced by human ALS-FUS expression [73,83]. In these studies, the knockdown of PRMT1 ortholog was done in transgenic flies already expressing human ALS-FUS. Therefore, the timing of inhibition may be the reason for the increased severity of the neurodegenerative phenotype.

Altogether, the opposing effect of PRMT1 knockdown in the cytoplasmic RNP granule formation of ALS-FUS is at first confusing, but most data seem to fit the model of PRMTs having a dual role in both nuclear import and LLPS of FUS. Inhibition of general methyltransferase activity or PRMT1 specifically rescues nuclear import of ALS-FUS by revealing alternative binding domain against transportin. However, the same treatment can also worsen the pathological phenotype of ALS-FUS if it is done after ALS-FUS had already been accumulated in the cytoplasm by promoting LLPS of FUS. These data underscore the potential of PRMT1 as a therapeutic target of ALS, as well as the need for caution in utilizing PRMTs as a therapeutic target.

### 5.2. Transcriptional Regulation

In NMD patients, the expression of genes related to inflammation, apoptosis, myelination, and axonal transport has shown to be altered, particularly in the early stages of the diseases [84,85]. PRMTs have a wide range of substrates, which include both histones and non-histone proteins, to regulate the gene expression profile. For example, histone H4 arginine 3 (H4R3) is a substrate of PRMT1, and asymmetric dimethylation of H4R3 has been shown to recruit histone acetyltransferases to activate various gene expression [86]. On the other hand, demethylation of H4R3 is associated with heterochromatin formation [86]. PRMT5 also methylates H4R3 alongside with histone H2A arginine 3, which leads to repression of set of genes including MYC target genes [24,87]. PRMT4 has shown to interact with p160 as a coactivator [24], while also functioning as a corepressor by blocking the interaction between cAMP response element-binding proteins (CREB) and CREB binding proteins [88]. The transcriptional regulatory role of PRMTs is suggested to be another mechanism for the enzymes to be involved in the progression of NMDs.

MS and MG are NMDs caused by autoimmune response that damages CNS and NMJ proteins. MS is characterized by damaged myelination and axons in the central nervous system, which leads to impaired vision, movement, fatigue, and muscle atrophy due to the misuse of the muscles. It usually occurs in adults of 20 to 45 years of age [89], and about 2.5 million individuals are affected worldwide [90]. MG is caused by autoantibodies attacking the acetylcholine related proteins in the postsynaptic membrane of muscle cells. This damages the NMJs, which in turn weakens the skeletal muscle. MG is reported to affect about 40–180 per million people worldwide [91], making it one of the major diseases targeting the NMJs. Studies suggest that PRMTs have important roles in immune cells through transcriptional regulation. Both PRMT1 and PRMT5 are found to be highly expressed in T helper cells, and global inhibition of methyltransferase activity showed an immunosuppressive effect on T cells [92,93]. PRMT5 in particular was found to be upregulated during T helper cell proliferation, and PRMT5 inhibition was sufficient to suppress memory T cell expansion [92]. A mechanistic study suggested that PRMT5 regulates the expression of IL-2 in T cells, which is important for the inflammatory response. While the direct target of PRMT5 that leads to the regulation of IL-2 expression has not been validated, several proteins involved in alternative splicing following T cell activation are found to be methylated by PRMT5 and proposed to be the potential link [94]. The deletion of PRMT5 in CD4^+^ T helper cells was able to suppress the inflammatory response and clinical signs of experimental autoimmune encephalomyelitis (EAE) mice, which is a model of MS [95]. 

Polyglutamine-expansion is an expansion of the CAG trinucleotide repeat that occurs in various genes and it is associated with a group of neurodegenerative diseases referred to as polyglutamine diseases. Androgen receptor (AR) is one of the genes that goes through polyglutamine-expansion to cause spinal and bulbar muscular atrophy (SBMA), which is a hereditary NMD that affects the lower motor neurons. The expanded polyglutamine sequence of AR is suggested to confer cytotoxicity mainly through a toxic gain of function, but the exact etiology has not been demonstrated [96]. The increased native transactivating function of AR due to polyglutamine expansion is another proposed mechanism of SBMA [97]. PRMT6 has been found to act as a coactivator of AR by methylating the Akt consensus site motif of AR, and this activity was shown to be enhanced by the polyglutamine-expansion [98]. As a result, inhibition of PRMT6 suppressed the neurodegenerative phenotype in the eye of drosophila caused by polyglutamine-expanded AR [98]. The data suggest the potential involvement of PRMT6 in SBMA etiology by promoting AR-mediated transactivation.

Cytoplasmic RNP granules sequester various proteins and RNAs from the site where they normally reside and function. For example, cytoplasmic aggregation of FUS leads to sequestration of PRMT1 from the nucleus [99]. These cells display demethylated H4R3, which is a sign of heterochromatin. The demethylated H4R3 recruits histone deacetylases to reduce acetylation of H3K9/K17, further expanding the heterochromatin histone marks [86,99]. This chromatin remodeling due to loss of nuclear function of PRMT1 can have widespread impact via transcriptional regulation of many genes. A study showed that the ALS-associated FUS-R521C mutant binds to PRMT1 more strongly than wild type FUS or other FUS mutants, sequestering the highest level of PRMT1 from the nucleus [82]. Furthermore, the FUS-R521C mutant expression in cortical neurons made the cells more susceptible to oxidative stress than other FUS mutants, manifested by the shorter length of neurites. The authors hypothesized that this increased susceptibility of cells against oxidative stress is due to the impaired nuclear function of PRMT1. Indeed, the overexpression of PRMT1 rescued the length of neurites under oxidative stress, while PRMT1 knockdown exacerbated the oxidative stress induced phenotype. Since PRMT1 has variety of ways to affect the cells, such as direct regulation of the stress granule formation as we discussed in the previous section, further examination would be required to show that the rescue is indeed due to the restoration of nuclear function of PRMT1. However, the differential affinity of a FUS mutant to PRMT1 suggests a potential mutation specific mechanism to induce or worsen disease phenotypes by interfering with the transcriptional regulatory function of PRMT1 in the nucleus. 

PRMT8 is particularly enriched in motor neurons and PRMT8 knockout mice exhibit muscle atrophy and NMJ fragmentation [100]. Transcriptome analysis revealed that the loss of PRMT8 decreases the expression of CREB1, disrupting the CREB1-dependent neuroprotective transcriptional network [100]. While more mechanistic studies are desired to delineate the downstream effectors and how they are modulated by PRMTs, these studies offer examples of PRMTs as a transcriptional regulator in association with NMDs.

### 5.3. Regulation of Protein-Protein and Protein-RNA Interactions

Spinal muscle atrophy (SMA) is an NMD characterized by degeneration of motor neurons and skeletal muscle due to mutations in the survival motor neuron 1 (SMN1) gene. The SMN protein is involved in pre-mRNA splicing and transport through binding to various splicing factors via its Tudor domain, which specifically recognizes and binds to methylated arginine and lysine residues [101]. Mutations in the Tudor domain of SMN have been shown to cause SMA, underscoring the significance of Tudor domain mediated binding activity of SMN in the disease progression [102]. Since SMN functions through selective binding against methylated arginine residues via its Tudor domain, PRMT may act as a potential mediator of the interaction between SMN and its binding partners. Indeed, many splicing factors such as CA150, SAP49, SmB, and U1C were shown to be substrates of PRMT4, and the interaction between CA150 and SMN was shown to be dependent on PRMT4 activity [103]. KH-type splicing regulatory protein (KSRP), which is another splicing factor that regulates mRNA decay in neurons, interacts with SMN and colocalizes in neurite granules in a PRMT4 dependent manner [104]. As KSRP bind and promote the decay of p21 mRNA, which is a negative cell cycle regulator that has been shown to be consistently upregulated in SMA patient models [105], the stabilization of p21 mRNA due to the loss of KSRP’s native function is proposed to be one of the contributors of the disease onset [104]. HuD is another splicing factor that functions through interaction with SMN and requires PRMT4 activity for the interaction [106]. p21 mRNA is also a target of HuD, but unlike KSRP, HuD stabilizes the p21 mRNA against decay [106]. Methylation of HuD by PRMT4 reduces the interaction between HuD and p21 mRNA and promotes decay of the mRNA [106]. These data support a preventive role of PRMT4 against SMA by promoting the decay of p21 mRNA. However, PRMT4 was shown to be upregulated at a protein level in SMA patient derived cells, and it is suggested to be a potential source of cytotoxicity through mis-regulation of alternative splicing and mRNA decay pathway [107,108]. While further studies would be needed to delineate the specific role of PRMT4 in SMA pathophysiology, the data strongly support the involvement of PRMT4 in the disease progression by modulating the Tudor domain binding sites of the splicing factors.

PRMT5 is another member of the PRMT family that is associated with SMA. Sm proteins are mis-localized in SMA patients, and localization of the proteins are regulated by symmetric dimethylation in their C-terminal tails by PRMT5 [109,110]. RNA polymerase II (RNAP II) is another substrate of PRMT5 and one of the binding partners of SMN via the Tudor domain. The interaction between RNAP II and SMN requires methylation of RNAP II R1810 by PRMT5, and successful interaction further recruits senataxin, an RNA-DNA helicase [111]. Together, this protein complex performs R-loop resolution during transcription. Mutations in senataxin are associated with ALS [112], likely due to dysfunctional R-loop resolution. Therefore, PRMT5 may also be a player in R-loop resolution associated ALS via the modulating interaction between SMN and RNAP II. 

The association of PRMTs in the progression of NMDs is continuously emerging. While some of the mechanisms of how PRMTs may be involved in the disease progression have been highlighted here, the readers should keep in mind that they are only a fraction of the many ways PRMTs can be involved in the process. Also, they are not mutually exclusive to one another, and the pathological phenotype is most likely a result of multiple mechanisms functioning simultaneously that eventually converge into the disease phenotype. This makes the use of PRMT as a therapeutic target very challenging, as modulating PRMT activity may incur numerous unintended consequences, or even an effect completely opposite to that intended. Therefore, therapeutic strategy targeting PRMT must account for as many variables and patient-specific conditions as possible.

## 6. Therapeutic Strategies

Our current understanding of the etiology of NMDs and how PRMTs are involved is limited. Here, we attempt to provide a broad guideline regarding the factors that needs to be accounted for when designing therapeutic strategies targeting PRMTs based on the studies to date. In the case of ALS, most of the patients exhibit cytoplasmic RNP granules that likely elicit cytotoxicity in motor neurons. Studies point to a dual role of PRMT1 in the formation of ALS-associated RNP granules. Inhibition of PRMT1 has shown to promote nuclear transport of ALS-FUS, but it can also facilitate LLPS of ALS-FUS to form cytoplasmic inclusions. To avoid unintended consequences, the state in which the patients are in regarding the ALS-FUS cytoplasmic localization at the time of treatment is critical. If the cytoplasmic concentration of FUS is already high, promoting PRMT activity may be beneficial to suppress LLPS of FUS by disrupting π-cation binding. On the other hand, PRMT1 inhibition may be utilized for cases where cytoplasmic content of FUS is still low, so that newly translated FUS mutants benefit from the rescued nuclear import. 

For MG and MS, targeting PRMT5 to suppress inflammatory response seems to be a promising measure. A potential side effect to be wary of in targeting PRMT5 is the trigger of apoptotic pathways by the downregulation of PRMT5. Downregulation of PRMT5 has shown to trigger apoptosis via the E2F-1/NF-kB/GSK-3b axis in various contexts [113,114]. Therefore, concurrent measures of antiapoptotic drugs alongside the PRMT5 inhibitor may be considered, or the dosage and duration of the PRMT inhibitor must be thoroughly tested and controlled.

DMD is a disease characterized by progressive muscle wasting due to a mutation in the dystrophin gene [115]. The dystrophin gene is located in the X chromosome and the X-linked recessive mutation affects about 1 in 5000 newborn boys [116]. The dystrophin protein functions as a structural stabilizer by connecting the muscle fibers to the extracellular matrix. Dysfunctional dystrophin induces chronic inflammation in the muscle fiber, which exhausts the muscle stem cells and interferes with the regeneration capacity of the muscle fibers. Gene therapy and stem cell therapy to restore the function of dystrophin in muscle cells are being developed, but restoration of already lost muscle mass would be a challenge [117]. Studies have suggested that a slow oxidative muscle type has a higher tolerance against disease progression [29,118]. In this regard, promoting PRMT7 activity may be a type of strategy to take to support muscle remodeling toward slow oxidative myofibers.

What must be considered for any strategies targeting PRMTs is to not disrupt the minimal level of PRMT activity required for cell survival, as the function of PRMTs, particularly PRMT1, has shown to be important for normal physiological functioning of the cells. As with any type of treatment, targeted delivery to a specific type of cells would be a safer option than a general treatment when targeting PRMTs. Also, continuous mechanistic study of both NMD etiology and PRMTs’ function in the future may allow for the designing of more personalized treatment strategies based on the mutant type or nongenetic factors of the patient to avoid side effects. For example, a mutation in the C9orf72 gene is the one of the most common causes of familial ALS and frontotemporal dementia [119,120]. The mutation consists of an abnormal expansion of a repeated hexanucleotide sequence (GGGGCC) in the first intron of the C9orf72 gene [119,120]. Repeat-associated non-AUG translation of this expansion produces dipeptide repeat proteins (DRPs), and the arginine containing DRPs, polyGR and polyPR are consistently reported to be the most toxic [121,122]. Premasiri and colleagues demonstrated that asymmetric dimethylation of polyGR and polyPR by Type I PRMTs plays important roles in their cytotoxicity. Moreover, they showed that Type I PRMT inhibitors can abrogate toxicity produced by exogenous polyGR and polyPR challenge in NSC34 cells [123], suggesting that Type I PRMT inhibition is a potential therapeutic strategy specifically for polyGR and polyPR induced ALS patients. Other examples of targeting PRMTs and its effect on specific NMD phenotype in preclinical models are listed in Table 1 with details on the type of mutation and model system.

Following the development of selective PRMT inhibitors targeting a specific PRMT isoform and preclinical studies on their usage, several selective PRMT inhibitors have entered clinical trials. GSK3326595, a PRMT5 inhibitor, was the first molecule targeting PRMTs to enter the clinical trial on 2016. Since then, a total of nine clinical trials of PRMT inhibitors have been listed on the clinicaltrials.gov website (Table 2). All nine of them test the inhibitors against cancer and are in phase 1/phase 2. Besides GSK3368715, which is a type I PRMT inhibitor, all of the molecules undergoing clinical studies are PRMT5 inhibitors. This is due to strong preclinical evidence of PRMT5’s involvement in oncogenesis and success in the development of PRMT5-specific inhibitors [10]. Only one of the nine trials is listed as completed, but the result has yet to be posted. Considering the timeline of the rest of the clinical trials, which are estimated to be completed between 2022 and 2025, we will soon be able to obtain data on the safety and efficacy of PRMT inhibitors in humans. The process is in the very early stages of looking into the usage of PRMT inhibitors as therapeutics, and therefore many opportunities lie ahead. Clinical trials investigating other diseases including NMDs are expected to follow in the near future.

## 7. Conclusions

The cases of neurodegenerative and neuromuscular diseases are continuously increasing, and they are projected to increase further in the future due to the aging population. However, we are many steps away from even identifying the cause of many NMDs. The relatively slow progress in this field signals the need for a different approach, perhaps transferring more attention toward nongenetic factors that may contribute to the diseases. PRMT biology seems to be one of the factors that deserves more attention. The significance of PRMTs in various diseases is constantly emerging, and NMDs are not an exception. What is intriguing in the PRMT biology is that the nine members of the PRMT family seem to work together to function as an intricate regulatory machine to maintain the homeostasis of the arginine methylation state in cells. There seem to be multiple layers of regulatory mechanisms through functional redundancy, substrate specificity, and direct regulation of each another to maintain the balance. While enzymes that function as arginine demethylase in vivo have not been confirmed, dynamic properties of the modification clearly seems to exist in terms of overall methylation level, the type of methylation (mono-, symmetric, asymmetric), and their distribution. For example, the inhibition of the major type I arginine methyltransferase, PRMT1, leads to an increase in the global MMA and SDMA level, giving us a peek into the competition that exists among different types of PRMTs for arginine methylation sites [124]. The pathological phenotype seems to be caused when this balance is disrupted. Both increased and decreased levels of PRMTs have been associated with various cancers and cardiovascular disease [25,125]. This characteristic of PRMT biology is consistent with the model describing the onset of NMDs in which “a pre-existing genetic load is acted on by time and environmental exposures until a tipping point is reached [126].” Further study on the implications of changes in the arginine methylation state of the proteome might offer the key to overcome NMDs.

## Figures and Tables

**Table 1:** Effect of targeting PRMTs on NMD phenotypes.

PRMT	Method	Model	Effect on NMD Phenotype
General methyltransferase inhibitor	AdOx	Hela cells	Rescues nuclear import of FUS mutants (R524S, R522G, R525L) [64]
General methyltransferase inhibitor	AdOx	Primary rat hippocampal neurons	Rescues nuclear import of FUS mutant (P525L) [64]
General methyltransferase inhibitor	AdOx	Primary motor neurons	Diminishes cytoplasmic FUS mutants (R521H, R521G, R521C) [72]
General methyltransferase inhibitor	AdOx	ALS patient-derived lymphoblastoid cells	Rescues nuclear import of FUS mutant (R518G) [73]
PRMT1	siRNA KD	Hela cells	Partial rescue of nuclear import of FUS mutant (P525L) [64]
PRMT1	KO	MEF	Diminishes cytoplasmic FUS mutants (R521H, R521G, R521C) [72]
PRMT1	siRNA KD	HEK293	Diminishes cytoplasmic FUS mutants (R521H, R521G, R521C) [72]
PRMT1	siRNA KD	Primary motor neurons	Increases cytoplasmic FUS mutants (R521H, R521G, R521C) [72]
PRMT1	Inhibitor (AMI-1)	ALS patient-derived lymphoblastoid cells	Rescues nuclear import of FUS mutant (R518G) [73]
PRMT1	shRNA KD	Cortical neurons	Enhances neurite shortening by FUS-R521C under oxidative stress [82]
PRMT1	Overexpression	Cortical neurons	Prevents neurite shortening by FUS-R521C under oxidative stress [82]
PRMT1	Inhibitor (MS023)	NSC-34	Abrogates PR_15_-induced toxicity [123]
DART1 (PRMT1/PRMT8 ortholog)	siRNA KD	Drosophila	Enhances neurodegeneration of eyes induced by wild-type FUS or FUS-R521H [73]
DART1 (PRMT1/PRMT8 ortholog)	siRNA KD	Drosophila	Enhances neurodegeneration of eyes induced by wild-type FUS or FUS-P525L [83]
PRMT5	Inhibitor (CMP5 or HLCL65)	Mouse memory T cells	Suppresses memory T cell expansion [92]
PRMT5	Inhibitor (CMP5) or shRNA KD	Human memory T cells	Suppresses memory T cell activation and expansion, partly through downregulation of IL-2 [92]
PRMT5	Inhibitor (CMP5)	OVA-induced DTH mouse	Suppresses T cell-mediated inflammatory response [92]
PRMT5	Inhibitor (HLCL65)	MOG-induced EAE mouse	Suppresses clinical signs of EAE through diminishing T cell-mediated inflammatory response [92]
PRMT5	CD4^+^ T-cell specific KO	MOG-induced EAE mouse	Suppresses clinical signs of EAE through diminishing T cell-mediated inflammatory response [95]
PRMT6	Overexpression	MN-1	Exacerbates cytotoxicity due to polyglutamine-expanded AR [98]
DART8 (PRMT6 ortholog)	RNAi KD	Drosophila	Suppresses neurodegenerative phenotype due to polyglutamine-expanded AR [98]

**Table 2:** Ongoing clinical trials of molecules targeting PRMTs.

Drug	Description	Phase	Disease	ClnicalTrials.gov Identifier	Study Date
GSK3326595	PRMT5 inhibitor	Phase 1	Solid tumor Lymphoma	NCT02783300	30 August 2016–29 April 2025
		Phase 1/2	Neoplasm	NCT03614728	16 October 2018–23 April 2025
		Phase 2	Breast cancer	NCT04676516	21 March 2021–31 December 2022
JNJ-64619178	PRMT5 inhibitor	Phase 1	Neoplasm	NCT03573310	13 July 2018–30 December 2022
PRT543	PRMT5 inhibitor	Phase 1	Solid tumor Lymphoma Leukemia	NCT03886831	11 February 2019–11 August 2022
PF-06939999	PRMT5 inhibitor	Phase 1	Solid tumor	NCT03854227	14 March 2019–21 September 2023
PRT811	PRMT5 inhibitor	Phase 1	Solid tumor Lymphoma Glioma	NCT04089449	6 November 2019–October 2022
AMG 193	PRMT5 inhibitor	Phase 1/2	Solid tumor	NCT05094336	30 December 2021–13 November 2024
GSK3368715	Type I PRMT inhibitor	Phase 1	Neoplasm	NCT03666988	22 October 2018–4 March 2021

## References

[1] Rygiel K.A., Picard M., Turnbull D. (2016). The ageing neuromuscular system and sarcopenia: A mitochondrial perspective. J. Physiol..

[2] Chhetri J.K., de Souto Barreto P., Fougère B., Rolland Y., Vellas B., Cesari M. (2018). Chronic inflammation and sarcopenia: A regenerative cell therapy perspective. Exp. Gerontol..

[3] Biolo G., Cederholm T., Muscaritoli M. (2014). Muscle contractile and metabolic dysfunction is a common feature of sarcopenia of aging and chronic diseases: From sarcopenic obesity to cachexia. Clin. Nutr..

[4] Cohen S., Nathan J.A., Goldberg A.L. (2014). Muscle wasting in disease: Molecular mechanisms and promising therapies. Nat. Rev. Drug Discov..

[5] Bonaldo P., Sandri M. (2013). Cellular and molecular mechanisms of muscle atrophy. Dis. Model. Mech..

[6] Fanzani A., Conraads V.M., Penna F., Martinet W. (2012). Molecular and cellular mechanisms of skeletal muscle atrophy: An update. J. Cachex Sarcopenia Muscle.

[7] Paik W.K., Kim S. (1968). Protein methylase I. Purification and properties of the enzyme. J. Biol. Chem..

[8] Bedford M.T., Clarke S.G. (2009). Protein Arginine Methylation in Mammals: Who, What, and Why. Mol. Cell.

[9] Pal S., Sif S. (2007). Interplay between chromatin remodelers and protein arginine methyltransferases. J. Cell. Physiol..

[10] Yang Y., Bedford M.T. (2013). Protein arginine methyltransferases and cancer. Nat. Rev. Cancer.

[11] Feng Y., Maity R., Whitelegge J.P., Hadjikyriacou A., Li Z., Zurita-Lopez C., Al-Hadid Q., Clark A.T., Bedford M.T., Masson J.Y. (2013). Mammalian protein arginine methyltransferase 7 (PRMT7) specifically targets RXR sites in lysine- and arginine-rich regions. J. Biol. Chem..

[12] Tang J., Kao P.N., Herschman H.R. (2000). Protein-arginine Methyltransferase I, the Predominant Protein-arginine Methyltransferase in Cells, Interacts with and Is Regulated by Interleukin Enhancer-binding Factor 3. J. Biol. Chem..

[13] Thandapani P., O’Connor T.R., Bailey T.L., Richard S. (2013). Defining the RGG/RG Motif. Mol. Cell.

[14] Blanc R.S., Richard S. (2017). Arginine Methylation: The Coming of Age. Mol. Cell.

[15] Thompson P.R., Fast W. (2006). Histone citrullination by protein arginine deiminase: Is arginine methylation a green light or a roadblock?. ACS Chem. Biol..

[16] Chang B., Chen Y., Zhao Y., Bruick R.K. (2007). JMJD6 is a histone arginine demethylase. Science.

[17] Stouth D.W., VanLieshout T.L., Shen N.Y., Ljubicic V. (2017). Regulation of Skeletal Muscle Plasticity by Protein Arginine Methyltransferases and Their Potential Roles in Neuromuscular Disorders. Front. Physiol..

[18] Pawlak M.R., Scherer C.A., Chen J., Roshon M.J., Ruley H.E. (2000). Arginine N-methyltransferase 1 is required for early postimplantation mouse development, but cells deficient in the enzyme are viable. Mol. Cell. Biol..

[19] Tee W.W., Pardo M., Theunissen T.W., Yu L., Choudhary J.S., Hajkova P., Surani M.A. (2010). Prmt5 is essential for early mouse development and acts in the cytoplasm to maintain ES cell pluripotency. Genes Dev..

[20] Ying Z., Mei M., Zhang P., Liu C., He H., Gao F., Bao S. (2015). Histone Arginine Methylation by PRMT7 Controls Germinal Center Formation via Regulating Bcl6 Transcription. J. Immunol..

[21] Torres-Padilla M.-E., Parfitt D.-E., Kouzarides T., Zernicka-Goetz M. (2007). Histone arginine methylation regulates pluripotency in the early mouse embryo. Nature.

[22] Swiercz R., Cheng D., Kim D., Bedford M.T. (2007). Ribosomal Protein rpS2 Is Hypomethylated in PRMT3-deficient Mice. J. Biol. Chem..

[23] Wei H.-H., Fan X.-J., Hu Y., Tian X.-X., Guo M., Mao M.-W., Fang Z.-Y., Wu P., Gao S.-X., Peng C. (2021). A systematic survey of PRMT interactomes reveals the key roles of arginine methylation in the global control of RNA splicing and translation. Sci. Bull..

[24] Guccione E., Richard S. (2019). The regulation, functions and clinical relevance of arginine methylation. Nat. Rev. Mol. Cell Biol..

[25] Sibal L., Agarwal S.C., Home P.D., Boger R.H. (2010). The Role of Asymmetric Dimethylarginine (ADMA) in Endothelial Dysfunction and Cardiovascular Disease. Curr. Cardiol. Rev..

[26] Wu Q., Schapira M., Arrowsmith C.H., Barsyte-Lovejoy D. (2021). Protein arginine methylation: From enigmatic functions to therapeutic targeting. Nat. Rev. Drug Discov..

[27] Tewary S.K., Zheng Y.G., Ho M.C. (2019). Protein arginine methyltransferases: Insights into the enzyme structure and mechanism at the atomic level. Cell. Mol. Life Sci..

[28] Iwasaki H., Yada T. (2007). Protein arginine methylation regulates insulin signaling in L6 skeletal muscle cells. Biochem. Biophys. Res. Commun..

[29] Ljubicic V., Khogali S., Renaud J.-M., Jasmin B.J. (2012). Chronic AMPK stimulation attenuates adaptive signaling in dystrophic skeletal muscle. Am. J. Physiol. Physiol..

[30] Blanc R.S., Vogel G., Li X., Yu Z., Li S., Richard S. (2017). Arginine Methylation by PRMT1 Regulates Muscle Stem Cell Fate. Mol. Cell. Biol..

[31] Kawabe Y., Wang Y.X., McKinnell I.W., Bedford M.T., Rudnicki M.A. (2012). Carm1 regulates Pax7 transcriptional activity through MLL1/2 recruitment during asymmetric satellite stem cell divisions. Cell Stem Cell.

[32] Chen S.L., Loffler K.A., Chen D., Stallcup M.R., Muscat G.E.O. (2002). The coactivator-associated arginine methyltransferase is necessary for muscle differentiation: CARM1 coactivates myocyte enhancer factor-2. J. Biol. Chem..

[33] Zhang T., Günther S., Looso M., Künne C., Krüger M., Kim J., Zhou Y., Braun T. (2015). Prmt5 is a regulator of muscle stem cell expansion in adult mice. Nat. Commun..

[34] Dacwag C.S., Ohkawa Y., Pal S., Sif S., Imbalzano A.N. (2007). The Protein Arginine Methyltransferase Prmt5 Is Required for Myogenesis because It Facilitates ATP-Dependent Chromatin Remodeling. Mol. Cell. Biol..

[35] Blanc R.S., Vogel G., Chen T., Crist C., Richard S. (2016). PRMT7 Preserves Satellite Cell Regenerative Capacity. Cell Rep..

[36] Jeong H.-J., Lee S.-J., Lee H.-J., Kim H.-B., Vuong T.A., Cho H., Bae G.-U., Kang J.-S. (2019). Prmt7 promotes myoblast differentiation via methylation of p38MAPK on arginine residue 70. Cell Death Differ..

[37] Choi S., Jeong H.-J., Kim H., Choi D., Cho S.-C., Seong J.K., Koo S.-H., Kang J.-S. (2019). Skeletal muscle-specific Prmt1 deletion causes muscle atrophy via deregulation of the PRMT6-FOXO3 axis. Autophagy.

[38] Shin H.-J.R., Kim H., Oh S., Lee J.-G., Kee M., Ko H.-J., Kweon M.-N., Won K.-J., Baek S.H. (2016). AMPK–SKP2–CARM1 signalling cascade in transcriptional regulation of autophagy. Nature.

[39] Liu Y., Li J., Shang Y., Guo Y., Li Z. (2018). CARM1 contributes to skeletal muscle wasting by mediating FoxO3 activity and promoting myofiber autophagy. Exp. Cell Res..

[40] Wang S.C.M., Dowhan D.H., Eriksson N.A., Muscat G.E.O. (2012). CARM1/PRMT4 is necessary for the glycogen gene expression programme in skeletal muscle cells. Biochem. J..

[41] Jeong H.-J., Lee H.-J., Vuong T.A., Choi K.-S., Choi D., Koo S.-H., Cho S.C., Cho H., Kang J.-S. (2016). Prmt7 Deficiency Causes Reduced Skeletal Muscle Oxidative Metabolism and Age-Related Obesity. Diabetes.

[42] Lin M., Xiong W.-C., Mei L. (2018). Neuromuscular Junction Formation, Aging, and Disorders. Annu. Rev. Physiol..

[43] Li X.M., Dong X.P., Luo S.W., Zhang B., Lee D.H., Ting A.K.L., Neiswender H., Kim C.H., Carpenter-Hyland E., Gao T.M. (2008). Retrograde regulation of motoneuron differentiation by muscle beta-catenin. Nat. Neurosci..

[44] Kakimoto Y., Matsuoka Y., Miyake M., Konishi H. (1975). Methylated amino acid residues of proteins of brain and other organs. J. Neurochem..

[45] Hashimoto M., Murata K., Ishida J., Kanou A., Kasuya Y., Fukamizu A. (2016). Severe Hypomyelination and Developmental Defects Are Caused in Mice Lacking Protein Arginine Methyltransferase 1 (PRMT1) in the Central Nervous System. J. Biol. Chem..

[46] Bezzi M., Teo S.X., Muller J., Mok W.C., Sahu S.K., Vardy L.A., Bonday Z.Q., Guccione E. (2013). Regulation of constitutive and alternative splicing by PRMT5 reveals a role for Mdm4 pre-mRNA in sensing defects in the spliceosomal machinery. Genes Dev..

[47] Chittka A., Nitarska J., Grazini U., Richardson W.D. (2012). Transcription Factor Positive Regulatory Domain 4 (PRDM4) Recruits Protein Arginine Methyltransferase 5 (PRMT5) to Mediate Histone Arginine Methylation and Control Neural Stem Cell Proliferation and Differentiation. J. Biol. Chem..

[48] Huang J., Vogel G., Yu Z., Almazan G., Richard S. (2011). Type II Arginine Methyltransferase PRMT5 Regulates Gene Expression of Inhibitors of Differentiation/DNA Binding Id2 and Id4 during Glial Cell Differentiation*. J. Biol. Chem..

[49] Fujiwara T., Mori Y., Chu D.L., Koyama Y., Miyata S., Tanaka H., Yachi K., Kubo T., Yoshikawa H., Tohyama M. (2006). CARM1 Regulates Proliferation of PC12 Cells by Methylating HuD. Mol. Cell. Biol..

[50] Selvi B.R., Swaminathan A., Maheshwari U., Nagabhushana A., Mishra R., Kundu T.K. (2015). CARM1 regulates astroglial lineage through transcriptional regulation of Nanog and posttranscriptional regulation by miR92a. Mol. Biol. Cell.

[51] Penney J., Seo J., Kritskiy O., Elmsaouri S., Gao F., Pao P.-C., Su S.C., Tsai L.-H. (2017). Loss of Protein Arginine Methyltransferase 8 Alters Synapse Composition and Function, Resulting in Behavioral Defects. J. Neurosci..

[52] Lee P.K.M., Bin Goh W.W., Sng J.C.G. (2017). Network-based characterization of the synaptic proteome reveals that removal of epigenetic regulatorPrmt8restricts proteins associated with synaptic maturation. J. Neurochem..

[53] Kim H.J., Jeong M.H., Kim K.R., Jung C.Y., Lee S.Y., Kim H., Koh K., Vuong T.A., Jung S.M., Yang H.W. (2016). Protein arginine methylation facilitates KCNQ channel-PIP2 interaction leading to seizure suppression. Elife.

[54] Lee S.-Y., Vuong T.A., Wen X., Jeong H.-J., So H.-K., Kwon I., Kang J.-S., Cho H. (2019). Methylation determines the extracellular calcium sensitivity of the leak channel NALCN in hippocampal dentate granule cells. Exp. Mol. Med..

[55] Statland J.M., Barohn R.J., McVey A.L., Katz J.S., Dimachkie M.M. (2015). Patterns of Weakness, Classification of Motor Neuron Disease, and Clinical Diagnosis of Sporadic Amyotrophic Lateral Sclerosis. Neurol. Clin..

[56] Ikenaka K., Atsuta N., Maeda Y., Hotta Y., Nakamura R., Kawai K., Yokoi D., Hirakawa A., Taniguchi A., Morita M. (2019). Increase of arginine dimethylation correlates with the progression and prognosis of ALS. Neurology.

[57] Chio A., Logroscino G., Hardiman O., Swingler R., Mitchell D., Beghi E., Traynor B.G., Eurals Consortium (2009). Prognostic factors in ALS: A critical review. Amyotroph. Lateral Scler..

[58] Brown R.H., Al-Chalabi A. (2017). Amyotrophic Lateral Sclerosis. N. Engl. J. Med..

[59] Longinetti E., Fang F. (2019). Epidemiology of amyotrophic lateral sclerosis: An update of recent literature. Curr. Opin. Neurol..

[60] Ling S.-C. (2018). Synaptic Paths to Neurodegeneration: The Emerging Role of TDP-43 and FUS in Synaptic Functions. Neural Plast..

[61] Fujii R., Takumi T. (2005). TLS facilitates transport of mRNA encoding an actin-stabilizing protein to dendritic spines. J. Cell Sci..

[62] Tan A.Y., Manley J.L. (2009). The TET Family of Proteins: Functions and Roles in Disease. J. Mol. Cell Biol..

[63] Dormann D., Rodde R., Edbauer D., Bentmann E., Fischer I., Hruscha A., Than M.E., Mackenzie I.R.A., Capell A., Schmid B. (2010). ALS-associated fused in sarcoma (FUS) mutations disrupt Transportin-mediated nuclear import. Embo. J..

[64] Dormann D., Rodde R., Edbauer D., Bentmann E., Fischer I., Hruscha A., Than M.E., Mackenzie I.R.A., Capell A., Schmind B. (2012). Arginine methylation next to the PY-NLS modulates Transportin binding and nuclear import of FUS. EMBO J..

[65] Gal J., Zhang J., Kwinter D.M., Zhai J., Jia H., Jia J., Zhu H. (2011). Nuclear localization sequence of FUS and induction of stress granules by ALS mutants. Neurobiol. Aging.

[66] Kwiatkowski T.J., Bosco D.A., Leclerc A.L., Tamrazian E., Vanderburg C.R., Russ C., Davis A., Gilchrist J., Kasarskis E.J., Munsat T. (2009). Mutations in the FUS/TLS Gene on Chromosome 16 Cause Familial Amyotrophic Lateral Sclerosis. Science.

[67] Winton M.J., Igaz L.M., Wong M.M., Kwong L.K., Trojanowski J.Q., Lee V.M.Y. (2008). Disturbance of nuclear and cytoplasmic TAR DNA-binding protein (TDP-43) induces disease-like redistribution, sequestration, and aggregate formation. J. Biol. Chem..

[68] Johnson B.S., Snead D., Lee J.J., McCaffrey M., Shorter J., Gitler A.D. (2009). TDP-43 is intrinsically aggregation-prone, and amyotrophic lateral sclerosis-linked mutations accelerate aggregation and increase toxicity. J. Biol. Chem..

[69] Nonaka T., Kametani F., Arai T., Akiyama H., Hasegawa M. (2009). Truncation and pathogenic mutations facilitate the formation of intracellular aggregates of TDP-43. Hum. Mol. Genet..

[70] Holt C.E., Schuman E.M. (2013). The Central Dogma Decentralized: New Perspectives on RNA Function and Local Translation in Neurons. Neuron.

[71] Rappsilber J., Friesen W.J., Paushkin S., Dreyfuss G., Mann M. (2003). Detection of Arginine Dimethylated Peptides by Parallel Precursor Ion Scanning Mass Spectrometry in Positive Ion Mode. Anal. Chem..

[72] Tradewell M.L., Yu Z., Tibshirani M., Boulanger M.-C., Durham H.D., Richard S. (2011). Arginine methylation by PRMT1 regulates nuclear-cytoplasmic localization and toxicity of FUS/TLS harbouring ALS-linked mutations. Hum. Mol. Genet..

[73] Scaramuzzino C., Monaghan J., Milioto C., Jr N.A.L., Maltare A., Aggarwal T., Casci I., Fackelmayer F.O., Pennuto M., Pandey U.B. (2013). Protein Arginine Methyltransferase 1 and 8 Interact with FUS to Modify Its Sub-Cellular Distribution and Toxicity In Vitro and In Vivo. PLoS ONE.

[74] Suárez-Calvet M., Neumann M., Arzberger T., Abou-Ajram C., Funk E., Hartmann H., Edbauer D., Kremmer E., Göbl C., Resch M. (2016). Monomethylated and unmethylated FUS exhibit increased binding to Transportin and distinguish FTLD-FUS from ALS-FUS. Acta Neuropathol..

[75] Kang J., Lim L., Lu Y., Song J. (2019). A unified mechanism for LLPS of ALS/FTLD-causing FUS as well as its modulation by ATP and oligonucleic acids. PLOS Biol..

[76] Wang J., Choi J.-M., Holehouse A.S., Lee H.O., Zhang X., Jahnel M., Maharana S., Lemaitre R., Pozniakovsky A., Drechsel D. (2018). A Molecular Grammar Governing the Driving Forces for Phase Separation of Prion-like RNA Binding Proteins. Cell.

[77] Evich M., Stroeva E., Zheng Y.G., Germann M.W. (2016). Effect of methylation on the side-chain pKa value of arginine. Protein Sci..

[78] Hofweber M., Hutten S., Bourgeois B., Spreitzer E., Niedner-Boblenz A., Schifferer M., Ruepp M.-D., Simons M., Niessing D., Madl T. (2018). Phase Separation of FUS Is Suppressed by Its Nuclear Import Receptor and Arginine Methylation. Cell.

[79] Qamar S., Wang G., Randle S.J., Ruggeri F.S., Varela J.A., Lin J.Q., Phillips E.C., Miyashita A., Williams D., Strohl F. (2018). FUS Phase Separation Is Modulated by a Molecular Chaperone and Methylation of Arginine Cation-π Interactions. Cell..

[80] Nott T., Petsalaki E., Farber P., Jervis D., Fussner E., Plochowietz A., Craggs T., Bazett-Jones D.P., Pawson T., Forman-Kay J.D. (2015). Phase Transition of a Disordered Nuage Protein Generates Environmentally Responsive Membraneless Organelles. Mol. Cell.

[81] Ryan V., Dignon G.L., Zerze G.H., Chabata C.V., Silva R., Conicella A.E., Amaya J., Burke K.A., Mittal J., Fawzi N.L. (2018). Mechanistic View of hnRNPA2 Low-Complexity Domain Structure, Interactions, and Phase Separation Altered by Mutation and Arginine Methylation. Mol. Cell.

[82] Jun M.H., Ryu H.H., Jun Y.W., Liu T., Li Y., Lim C.S., Lee Y.S., Kaang B.K., Jang D.J., Lee J.A. (2017). Sequestration of PRMT1 and Nd1-L mRNA into ALS-linked FUS mutant R521C-positive aggregates contributes to neurite degeneration upon oxidative stress. Sci. Rep..

[83] Jäckel S., Summerer A.K., Thömmes C.M., Pan X., Voigt A., Schulz J.B., Rasse T.M., Dormann D., Haass C., Kahle P.J. (2015). Nuclear import factor transportin and arginine methyltransferase 1 modify FUS neurotoxicity in Drosophila. Neurobiol. Dis..

[84] Oliveira G.P., Alves C.J., Chadi G. (2013). Early gene expression changes in spinal cord from SOD1G93A Amyotrophic Lateral Sclerosis animal model. Front. Cell. Neurosci..

[85] Scekic-Zahirovic J., Sendscheid O., El Oussini H., Jambeau M., Sun Y., Mersmann S., Wagner M., Dieterlé S., Sinniger J., Dirrig-Grosch S. (2016). Toxic gain of function from mutant FUS protein is crucial to trigger cell autonomous motor neuron loss. EMBO J..

[86] Huang S., Litt M., Felsenfeld G. (2005). Methylation of histone H4 by arginine methyltransferase PRMT1 is essential in vivo for many subsequent histone modifications. Genes Dev..

[87] Scaglione A., Patzig J., Liang J., Frawley R., Bok J., Mela A., Yattah C., Zhang J., Teo S.X., Zhou T. (2018). PRMT5-mediated regulation of developmental myelination. Nat. Commun..

[88] Xu W., Chen H., Du K., Asahara H., Tini M., Emerson B.M., Montminy M., Evans R.M. (2001). A Transcriptional Switch Mediated by Cofactor Methylation. Science.

[89] Brust J.C. (2018). Current Diagnosis & Treatment Neurology.

[90] Hauser S.L., Goodin D.S., Kasper D.L., Fauci A.S., Hauser S.L., Longo D.L., Jameson J.L., Loscalzo J. (2014). Multiple Sclerosis and Other Demyelinating Diseases. Harrison’s Principles of Internal Medicine, 19e.

[91] Heldal A.T., Owe J.F., Gilhus N.E., Romi F. (2009). Seropositive myasthenia gravis: A nationwide epidemiologic study. Neurology.

[92] Webb L.M., Amici S.A., Jablonski K.A., Savardekar H., Panfil A.R., Li L., Zhou W., Peine K., Karkhanis V., Bachelder E.M. (2017). PRMT5-Selective Inhibitors Suppress Inflammatory T Cell Responses and Experimental Autoimmune Encephalomyelitis. J. Immunol..

[93] Parry R.V., Ward S.G. (2010). Protein arginine methylation: A new handle on T lymphocytes?. Trends Immunol..

[94] Sengupta S., West K.O., Sanghvi S., Laliotis G., Agost L.M., Lynch K.W., Tsichlis P.N., Singh H., Patrick K.L., Guerau-de-Arellano M. (2021). PRMT5 Promotes Symmetric Dimethylation of RNA Processing Proteins and Modulates Activated T Cell Alternative Splicing and Ca^2+^/NFAT Signaling. Immunohorizons.

[95] Webb L.M., Sengupta S., Edell C., Piedra-Quintero Z.L., Amici S.A., Janiret Narvaez M., Bevins M., Kennemer A., Laliotis G., Tsichlis P.N. (2020). Protein arginine methyltransferase 5 promotes cholesterol biosynthesis–mediated Th17 responses and autoimmunity. J. Clin. Investig..

[96] Adachi H., Waza M., Katsuno M., Tanaka F., Doyu M., Sobue G. (2007). Pathogenesis and molecular targeted therapy of spinal and bulbar muscular atrophy. Neuropathol. Appl. Neurobiol..

[97] Orr H.T. (2012). Polyglutamine neurodegeneration: Expanded glutamines enhance native functions. Curr. Opin. Genet. Dev..

[98] Scaramuzzino C., Casci I., Parodi S., Lievens P.M., Polanco M.J., Milioto C., Chivet M., Monaghan J., Mishra A., Badders N. (2015). Protein Arginine Methyltransferase 6 Enhances Polyglutamine-Expanded Androgen Receptor Function and Toxicity in Spinal and Bulbar Muscular Atrophy. Neuron.

[99] Tibshirani M., Tradewell M.L., Mattina K.R., Minotti S., Yang W., Zhou H., Strong M., Hayward L.J., Durham H.D. (2014). Cytoplasmic sequestration of FUS/TLS associated with ALS alters histone marks through loss of nuclear protein arginine methyltransferase 1. Hum. Mol. Genet..

[100] Simandi Z., Pajer K., Karolyi K., Sieler T., Jiang L.-L., Kolostyak Z., Sari Z., Fekecs Z., Pap A., Patsalos A. (2018). Arginine Methyltransferase PRMT8 Provides Cellular Stress Tolerance in Aging Motoneurons. J. Neurosci..

[101] Coady T.H., Lorson C.L. (2011). SMN in spinal muscular atrophy and snRNP biogenesis. Wiley Interdiscip. Rev. RNA.

[102] Côté J., Richard S. (2005). Tudor Domains Bind Symmetrical Dimethylated Arginines. J. Biol. Chem..

[103] Cheng D., Côté J., Shaaban S., Bedford M.T. (2007). The Arginine Methyltransferase CARM1 Regulates the Coupling of Transcription and mRNA Processing. Mol. Cell.

[104] Tadesse H., Deschênes-Furry J., Boisvenue S., Côté J. (2008). KH-type splicing regulatory protein interacts with survival motor neuron protein and is misregulated in spinal muscular atrophy. Hum. Mol. Genet..

[105] Olaso R., Joshi V., Fernandez J., Roblot N., Courageot S., Bonnefont J.P., Melki J. (2006). Activation of RNA metabolism-related genes in mouse but not human tissues deficient in SMN. Physiol. Genom..

[106] Hubers L., Valderrama-Carvajal H., LaFramboise J., Timbers J., Sanchez G., Côté J. (2010). HuD interacts with survival motor neuron protein and can rescue spinal muscular atrophy-like neuronal defects. Hum. Mol. Genet..

[107] Sanchez G., Dury A.Y., Murray L.M., Biondi O., Tadesse H., EL Fatimy R., Kothary R., Charbonnier F., Khandjian E.W., Côté J. (2012). A novel function for the survival motoneuron protein as a translational regulator. Hum. Mol. Genet..

[108] Sanchez G., Bondy-Chorney E., Laframboise J., Paris G., Didillon A., Jasmin B.J., Côté J. (2015). A novel role for CARM1 in promoting nonsense-mediated mRNA decay: Potential implications for spinal muscular atrophy. Nucleic Acids Res..

[109] Branscombe T.L., Frankel A., Lee J.H., Cook J.R., Yang Z., Pestka S., Clarke S. (2001). PRMT5 (Janus kinase-binding protein 1) catalyzes the formation of symmetric dimethylarginine residues in proteins. J. Biol. Chem..

[110] Boisvert F.M., Cote J., Boulanger M.C., Cleroux P., Bachand F., Autexier C., Richard S. (2002). Symmetrical dimethylarginine methylation is required for the localization of SMN in Cajal bodies and pre-mRNA splicing. J. Cell. Biol..

[111] Zhao D.Y., Gish G., Braunschweig U., Li Y., Ni Z., Schmitges F.W., Zhong G., Liu K., Li W., Moffat J. (2015). SMN and symmetric arginine dimethylation of RNA polymerase II C-terminal domain control termination. Nature.

[112] Hirano M., Quinzii C.M., Mitsumoto H., Hays A.P., Roberts J.K., Richard P., Rowland L.P. (2010). Senataxin mutations and amyotrophic lateral sclerosis. Amyotroph. Lateral Scler..

[113] Quan X., Yue W., Luo Y., Cao J., Wang H., Wang Y., Lu Z. (2015). The protein arginine methyltransferase PRMT5 regulates Aβ-induced toxicity in human cells and Caenorhabditis elegans models of Alzheimer’s disease. J. Neurochem..

[114] Li M., An W., Xu L., Lin Y., Su L., Liu X. (2019). The arginine methyltransferase PRMT5 and PRMT1 distinctly regulate the degradation of anti-apoptotic protein CFLAR(L) in human lung cancer cells. J. Exp. Clin. Cancer Res..

[115] Mercuri E., Bonnemann C.G., Muntoni F. (2019). Muscular dystrophies. Lancet.

[116] Mendell J.R., Lloyd-Puryear M. (2013). Report of MDA muscle disease symposium on newborn screening for Duchenne muscular dystrophy. Muscle Nerve.

[117] Verhaart I.E.C., Aartsma-Rus A. (2019). Therapeutic developments for Duchenne muscular dystrophy. Nat. Rev. Neurol..

[118] Mizobuchi M., Inoue R., Miyaka M., Kakimoto Y. (1985). Accelerated protein turnover in the skeletal muscle of dystrophic mice. Biochim. et Biophys. Acta Gen. Subj..

[119] DeJesus-Hernandez M., Mackenzie I.R., Boeve B.F., Boxer A.L., Baker M., Rutherford N.J., Nicholson A.M., Finch N.A., Flynn H., Adamson J. (2011). Expanded GGGGCC hexanucleotide repeat in noncoding region of C9ORF72 causes chromosome 9p-linked FTD and ALS. Neuron.

[120] Renton A.E., Majounie E., Waite A., Simon-Saánchez J., Rollinson S., Gibbs J.R., Schymick J.C., Laaksovirta H., van Swieten J.C., Myllykangas L. (2011). A Hexanucleotide Repeat Expansion in C9ORF72 Is the Cause of Chromosome 9p21-Linked ALS-FTD. Neuron.

[121] Freibaum B.D., Lu Y., Lopez-Gonzalez R., Kim N.C., Almeida S., Lee K.-H., Badders N., Valentine M., Miller B.L., Wong P.C. (2015). GGGGCC repeat expansion in C9orf72 compromises nucleocytoplasmic transport. Nature.

[122] Lee K.-H., Zhang P., Kim H.J., Mitrea D.M., Sarkar M., Freibaum B.D., Cika J., Coughlin M., Messing J., Molliex A. (2016). C9orf72 Dipeptide Repeats Impair the Assembly, Dynamics, and Function of Membrane-Less Organelles. Cell.

[123] Premasiri A., Gill A.L., Vieira F.G. (2020). Type I PRMT Inhibition Protects Against C9ORF72 Arginine-Rich Dipeptide Repeat Toxicity. Front. Pharmacol..

[124] Dhar S., Vemulapalli V., Patananan A.N., Huang G.L., Di Lorenzo A., Richard S., Comb M.J., Guo A., Clarke S.G., Bedford M.T. (2013). Loss of the major Type I arginine methyltransferase PRMT1 causes substrate scavenging by other PRMTs. Sci. Rep..

[125] Hwang J.W., Cho Y., Bae G.-U., Kim S.-N., Kim Y.K. (2021). Protein arginine methyltransferases: Promising targets for cancer therapy. Exp. Mol. Med..

[126] Al-Chalabi A., Hardiman O. (2013). The epidemiology of ALS: A conspiracy of genes, environment and time. Nat. Rev. Neurol..

