# Correction: Lee et al. Protein Arginine Methyltransferases in Neuromuscular Function and Diseases. *Cells* 2022, *11*, 364

**DOI:** 10.3390/cells11162609

**Published:** 2022-08-22

**Authors:** Jinwoo Lee, Subin An, Sang-Jin Lee, Jong-Sun Kang

**Affiliations:** 1 Research Institute for Aging-Related Diseases, AniMusCure Inc., Suwon 16419, Korea; 2 Department of Molecular Cell Biology, School of Medicine, Sungkyunkwan University, Suwon 16419, Korea; 3 Single Cell Network Research Center, Sungkyunkwan University, Suwon 16419, Korea

The authors wish to make the following corrections to this paper [1]:

For Table 1, reference [1] has been changed to [64]; reference [2] has been changed to [72]; reference [3] has been changed to [73]; reference [4] has been changed to [82]; reference [5] has been changed to [123]; reference [6] has been changed to [83]; reference [7] has been changed to [92]; reference [8] has been changed to [95]; reference [9] has been changed to [98]. The corrected Table 1 showed as below:

The authors apologize for any inconvenience caused and state that the scientific conclusions are unaffected. The original publication has also been updated.

## Figures and Tables

**Table 1:** Effect of targeting PRMTs on NMD phenotypes.

PRMT	Method	Model	Effect on NMD Phenotype
General methyltransferase inhibitor	AdOx	Hela cells	Rescues nuclear import of FUS mutants (R524S, R522G, R525L) [64]
General methyltransferase inhibitor	AdOx	Primary rat hippocampal neurons	Rescues nuclear import of FUS mutant (P525L) [64]
General methyltransferase inhibitor	AdOx	Primary motor neurons	Diminishes cytoplasmic FUS mutants (R521H, R521G, R521C) [72]
General methyltransferase inhibitor	AdOx	ALS patient-derived lymphoblastoid cells	Rescues nuclear import of FUS mutant (R518G) [73]
PRMT1	siRNA KD	Hela cells	Partial rescue of nuclear import of FUS mutant (P525L) [64]
PRMT1	KO	MEF	Diminishes cytoplasmic FUS mutants (R521H, R521G, R521C) [72]
PRMT1	siRNA KD	HEK293	Diminishes cytoplasmic FUS mutants (R521H, R521G, R521C) [72]
PRMT1	siRNA KD	Primary motor neurons	Increases cytoplasmic FUS mutants (R521H, R521G, R521C) [72]
PRMT1	Inhibitor (AMI-1)	ALS patient-derived lymphoblastoid cells	Rescues nuclear import of FUS mutant (R518G) [73]
PRMT1	shRNA KD	Cortical neurons	Enhances neurite shortening by FUS-R521C under oxidative stress [82]
PRMT1	Overexpression	Cortical neurons	Prevents neurite shortening by FUS-R521C under oxidative stress [82]
PRMT1	Inhibitor (MS023)	NSC-34	Abrogates PR_15_-induced toxicity [123]
DART1 (PRMT1/PRMT8 ortholog)	siRNA KD	Drosophila	Enhances neurodegeneration of eyes induced by wild-type FUS or FUS-R521H [73]
DART1 (PRMT1/PRMT8 ortholog)	siRNA KD	Drosophila	Enhances neurodegeneration of eyes induced by wild-type FUS or FUS-P525L [83]
PRMT5	Inhibitor (CMP5 or HLCL65)	Mouse memory T cells	Suppresses memory T cell expansion [92]
PRMT5	Inhibitor (CMP5) or shRNA KD	Human memory T cells	Suppresses memory T cell activation and expansion, partly through downregulation of IL-2 [92]
PRMT5	Inhibitor (CMP5)	OVA-induced DTH mouse	Suppresses T cell-mediated inflammatory response [92]
PRMT5	Inhibitor (HLCL65)	MOG-induced EAE mouse	Suppresses clinical signs of EAE through diminishing T cell-mediated inflammatory response [92]
PRMT5	CD4^+^ T-cell specific KO	MOG-induced EAE mouse	Suppresses clinical signs of EAE through diminishing T cell-mediated inflammatory response [95]
PRMT6	Overexpression	MN-1	Exacerbates cytotoxicity due to polyglutamine-expanded AR [98]
DART8 (PRMT6 ortholog)	RNAi KD	Drosophila	Suppresses neurodegenerative phenotype due to polyglutamine-expanded AR [98]
